# Favourable clinical outcomes following cemented arthroplasty after metal-on-metal total hip replacement: a retrospective study with a mean follow-up of 10 years

**DOI:** 10.1186/s12891-020-03797-y

**Published:** 2020-11-21

**Authors:** Weiguang Yu, Meiji Chen, Xianshang Zeng, Mingdong Zhao, Xinchao Zhang, Junxing Ye, Jintao Zhuang, Guowei Han

**Affiliations:** 1 Department of Orthopaedics, The First Affiliated Hospital, Sun Yat-sen University, No. 58, Zhongshan 2nd Road, Yuexiu District, Guangzhou, 510080 China; 2 Department of Pediatrics, The First Affiliated Hospital, Sun Yat-sen University, No. 58, Zhongshan 2nd Road, Yuexiu District, Guangzhou, 510080 China; 3 Department of Orthopaedics, Jinshan Hospital, Fudan University, Longhang Road No. 1508, Jinshan District, Shanghai, 201508 China; 4 Department of Orthopaedics, The Affiliated Hospital of Jiangnan University, No. 1000, Hefeng Road, Binhu District, Wuxi, 21400 Jiangsu China; 5 Department of Orthopaedics, The Third People’s Hospital of Wuxi, No. 1000, Hefeng Road, Binhu District, Wuxi, 214000 Jiangsu China; 6 Department of Urinary surgery, The First Affiliated Hospital, Sun Yat-sen University, No. 58, Zhongshan 2nd Road, Yuexiu District, Guangzhou, 510080 China

**Keywords:** Revision, Metal-on-metal, Total hip replacement, Outcome, Cemented

## Abstract

**Background:**

Given the unexpected high rate of failure following metal-on-metal total hip replacement (MoM-THR), it is expected that more MoM-THR patients will experience revision. The long-term outcomes regarding the primary MoM-THR revised to cemented THR (CTHR) remain controversial. The purpose of this retrospective review was to evaluate the long-term outcomes of patients who underwent conversion from MoM-THR to CTHR.

**Methods:**

A total of 220 patients (220 hips) who underwent a conversion of primary MoM-THR to CTHR from March 2006 to October 2016 were retrospectively reviewed. The primary outcomes were the functional outcomes assessed using the Harris hip scores (HHS) and major radiographic outcomes. Follow-ups occurred at 3 months, 6 months, 1 year, 2 years, and then every two years after revision.

**Results:**

Mean follow-up was 10.1 years (5–13 years). Distinct improvements were detected in the mean HHS between the preoperative and last follow-up analysis (62.35[±8.49] vs. 84.70[±14.68], respectively, *p* < 0.001). The key orthopaedic complication rate was 18.2% (27/148). Seven (4.7%) cases experienced a CTHR failure at a mean of 3.4 (±1.2) years after revision MoM-THR, mostly attributed to recurrent dislocation.

**Conclusion:**

CTHR might yield an acceptable functional score and a low rate of the key orthopaedic complications.

## Background

Approximately half of patients experiencing metal-on-metal total hip replacement (MoM-THR) subsequently received leading implant-related complications with more than 1/3 undertaking secondary revision surgery [1, 2]. Implant-related complications associated with adverse reactions to metal debris (ARMD) have been an increasing concern [3, 4]. The occurrence of these complications stimulated by ARMD which is forcefully implicated in the pathophysiology of MoM-THR failure is common and may be associated with osteolysis [5]. Furthermore, revision MoM-THR exposes to two leading challenges on the acetabular side especially when severe acetabular bone defect was reconstructed [6]. When reconstruction of acetabular bone defect related to pseudotumor was required, patients undergoing MoM-THR revised to cemented THR (CTHR) which was deemed to have the advantage of both increasing stability and persistent articulating bearing might still have secondary damage to the joints [7, 8]. To date, there remains a discrepancy of data regarding the long-term outcomes of MoM-THR revised to CTHR [9, 10].

We therefore reviewed our population of patients who underwent a conversion of primary MoM-THR to CTHR to assess the 10-year follow-up outcomes. We hypothesize that submitted revision total hip surgeries without metal would provide a reasonable salvage procedure in a mid to longer-term follow-up.

## Methods

### Study population

Two hundred and twenty consecutive patients who were treated with CTHR after primary MoM-THR failure in the First Affiliated Hospital, Sun Yat-sen University between March 2006 and October 2016 were retrospectively reviewed. Demographic data, time to conversion, Harris hip score (HHS) prior to revision, and mechanism of injury were obtained from medical records and radiographic review. The inclusion criteria were as follows: patients who experienced conversion from initial MoM-THR (Zimmer, Warsaw, Poland) to CTHR (stem and cup, Elite, Stryker, America) with a central cup hole which can ensure that the cup is rigidly attached to the acetabular shell. The exclusion criteria included lacking research data, uncemented and hybrid THRs, congenital or acquired hip dysplasia, neuromuscular disorders, malignant tumour, severe trauma, active bleeding, poor medical conditions (i.e., acute respiratory distress syndrome, multiple organ dysfunction syndrome, diabetic acidosis, and severe malnutrition), and dementia.

The indication for MoM-THR to CTHR conversion involved ARMD, recurrent dislocation, wear, and loosening. All the conversions of MoM-THR to CTHR were performed by four fellowship-trained orthopaedist (WY, XSZ, MZ, and XCZ) using a posterolateral approach with removal of all MoM-THR components and insertion of CTHR components. The acetabular prostheses were implanted using a press-fit technique with screws. The surgical details and postoperative management were consistent with our previous descriptions [11]. Clinical and radiological data were followed up. The patients were reviewed postoperatively at 3 months, 6 months, 12 months, 24 months by the surgeons. Subsequent reviews occurred at every two years. The primary outcomes were both the functional outcomes assessed using HHS which less than 70 was regarded as a failure and the key orthopaedic complications including implant failure, loosening, dislocation, heterotopic ossification (≥ grade 3), and periprosthesis fractures. The definition of femoral and acetabular loosening was a cup migration or angular rotation exceeding 3 mm or a continuous radiolucent line wider than 2 mm [12, 13]. Implant failure was defined as well-defined migration and eccentric wear of the cup [14]. Heterotopic ossification was assessed using Brooker’s classification [15]. Revision was defined as removal or exchange of any part of the prosthesis [16].

### Statistical analysis

Comparison of functional outcomes at each follow-up was performed per Student’s t-test. The date of revision for any cause was regarded as the date of implant failure. A *p*-value of less than 0.05 was used as a threshold for significance. The key statistical analyses were executed using SPSS, 26.0 (IBM Corp., Armonk, NY). Other statistical analyses were done through GraphPad Prism 8(GraphPad Software Inc., San Diego, CA, USA).

## Results

**Fig. 1:**
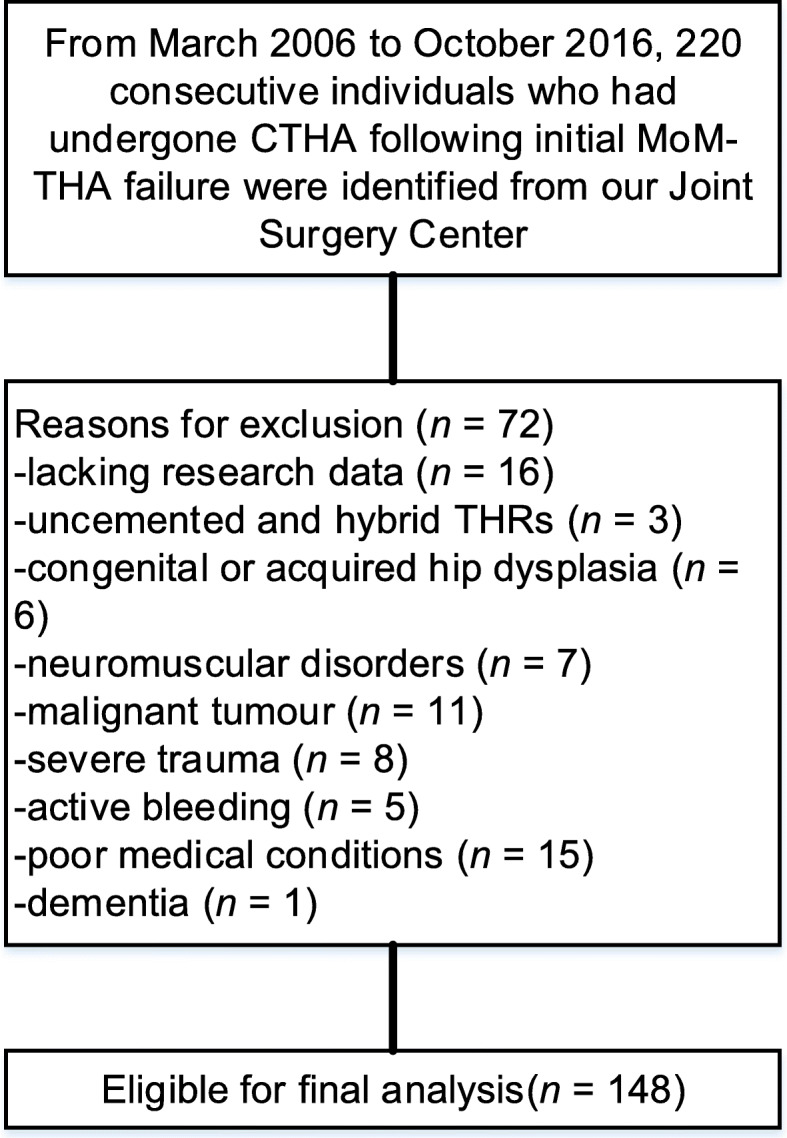
Flow diagram presenting the method for identification of individuals to evaluate the clinical and radiological outcomes of patients who had experienced a primary metal-on-metal total hip replacement (MoM-THR) revised to cemented THR (CTHR)

In total, 220 patients who underwent MoM-THR revised to CTHR were identified. Of these, 72 (32.7%) patients were excluded according to the current criteria, leaving 148 patients for final analysis (Fig. 1). The mean age of patients at the time of revision was 52.4 years (46–62 years). there was a male predominance (80 males vs. 68 females) in the cohort. The mean interval from failed MoM-THR to conversion was 6.8 years (2–9 years). The mean bone mineral density (BMD) was − 3.58(− 3.1 to − 3.9). The mean HHS prior to conversion was 57.71 (±13.85). The mean follow-up was 10.1 years (5–13 years). The baseline data were presented in Table 1.

**Table 1:** Baseline data on the overall population of 148 patients

Variable	CTHR (*n* = 148)
Sex, M/F	80/68
Age(y)	52.4 (46–62)
BMI (kg/m^2^)	26.7 (20–31)
BMD	−3.58 (− 3.1 to − 3.9)
Side, left/right	83/65
Time to conversion (y)	6.8 (2–9)
Comorbidities, n%	
Hypertension	49 (33.1)
Diabetes mellitus	31 (20.9)
Hypertension and Diabetes mellitus	12 (8.1)
Mechanism of injury, n%	
Traffic	34 (22.9)
Falling	69 (46.6)
Tamp	35 (23.6)
Other	10 (6.8)
ASA scale, n%	
I	43 (29.1)
II	75 (50.7)
III	30 (20.3)
HHS prior to revision	62.35 ± 8.49
Follow-up time (y)	10.1 (7–13)

*CTHR* cemented total hip replacement, *BMI* body mass index, *BMD* bone mineral density*, MoM-THR* metal-on-metal total hip replacement, *ASA* American Society of Anesthesiologists, *HHS* Harris hip scores

### Clinical outcomes

**Fig. 2:**
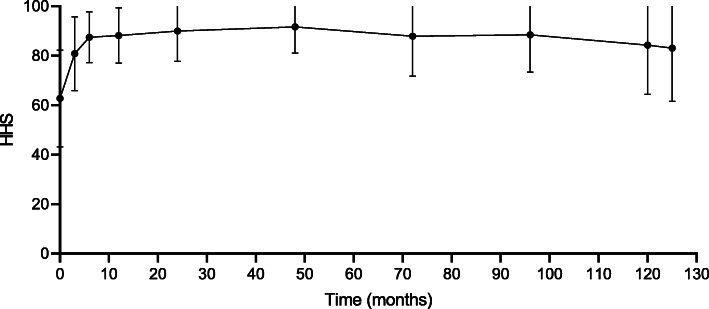
The variation trend of postoperative functional scores

The mean HHS after revision was presented in Table 2. Figure 2 showed the variation trend of postoperative functional scores. Noteworthy improvements were detected in the mean HHS between the preoperative and last follow-up assessments (62.35[±8.49] vs. 84.70[±14.68], respectively, *p* < 0.001), between 2 years after revision and 4 years after revision (88.76[±9.27] vs. 91.40[±10.59], respectively, *p* < 0.001), between 4 years after revision and 6 years after revision (91.40[±10.59] vs. 88.16[±10.02], respectively, *p* < 0.001), and between 6 years after revision and 8 years after revision (88.16[±10.02] vs. 87.35 ± 11.28, respectively, *p* < 0.001). The HHS peaked at 4 years after conversion (91.40[±10.59]). There was no significant drop off at the final follow-up starting with the fourth year after conversion.

**Table 2:** Clinical outcomes of patients undergoing a primary MoM-THR revised to CTHR

HHS, month(s) after conversion	CTHR (*n* = 148)
3	80.01 ± 9.75^a^
6	86.14 ± 6.31
12	88.52 ± 6.83
24	88.76 ± 9.27^a^
48	91.40 ± 10.59^a^
72	88.16 ± 10.02^a^
96	87.35 ± 11.28^a^
120	85.72 ± 13.65
Final follow-up	84.70 ± 14.68^a^

^a^Statistically significant values*. CTHR* cemented total hip replacement, *MoM-THR* metal-on-metal total hip replacement, *HHS* Harris hip scores

**Table 3:** Radiological outcomes of patients undergoing a primary MoM-THR revised to CTHR

Variable, n%	CTHR (*n* = 148)
Implant failure	7 (4.7)
Loosening	8 (5.4)
Dislocation	10 (6.8)
Heterotopic ossification (≥ grade 3)	0 (0.0)
Periprosthesis fracture	2 (1.4)

*CTHR* cemented total hip replacement, *MoM-THR* metal-on-metal total hip replacement

### Radiological outcomes

At the last review, 27 key orthopaedic complications in 148 cases were observed (Table 3). The rate of key orthopaedic complication was 18.2%. Seven (4.7%) cases experienced a CTHR failure at a mean of 4.4 years (3–6 years) after revision MoM-THR, mostly attributed to recurrent dislocation. Eight (5.4%) had a loosening at a mean of 2.6 (±1.1) years after conversion. Ten (6.8%) had a dislocation. Heterotopic ossification (≥ grade 3) failed to be detected throughout the follow-up period. Two (1.4%) had a periprosthesis fracture.

## Discussion

The current study shows that the conversion from primary MoM-THR to CTHR tends to have favourable clinical outcomes, involving an acceptable functional score and a low rate of the key orthopaedic complications. The acetabular component tends to have loss of acetabular bone stock after removal of MoM articulation [17]. As well, removal of the MoM articulation was associated with reducing metal ion levels [18]. The utilisation of a cemented bearing for revision MoM-THR theoretically contributes to decreasing the high associated dislocation rate [19]. Maybe, CTHR is a preferred option for management of patients with MoM-THR failure, although the impact of residual metal ions on CTHR and the extent of the impact are still unclear [20, 21].

Although use of large-diameter MoM articulations, potential drawbacks associated with MoM cups have been frequently reported in published literatures [3, 8, 22]. These drawbacks primarily involve ARMD, periarticular pseudotumor, and systemic complications related to metal ions, which restricts the further promotion of MoM-THR [8, 20]. MoM-THR with wear characteristics (i.e., fretting corrosion) releasing metal ions, stimulating the surrounding bone and tendon tissue, leading to osteoporosis and tendon tissue hyperplasia, which in turn triggers the instability after MoM-THR surgery has been regarded as a leading initiator for subsequent revision [23, 24]. When MoM-THR was revised to CTHR, such a dilemma related to wear characteristics (i.e., fretting corrosion) still exists [25]. Furthermore, disassociation of the femoral head from the stem following gross wear of the taper is common in patients who were treated with MoM-THR [7].

Previous reports [23, 24] have shown that the increased migration of CTHR revision following prior MoM-THR failure, which was associated with dislocation, especially if recurrent. Cemented components can improve osteoporosis to a certain extent [26]. However, it is unclear whether the damage triggered by the residual metal ions to bone continues after conversion, or whether there was a positive correlation between the CTHR stability and decreasing serum cobalt and chromium levels [18, 25], although several studies [27, 28] have shown that after the conversion of MoM-THR to CTHR, serum cobalt and chromium levels were reduced.

Decreased metal ion level, coupled with the potential to resist impingement results in improved symptoms initiated by ARMD in patients who experienced this conversion [28]. Lainiala et al. [29] reported approximately 2500 individuals who experienced a primary MoM-THR and revealed that 63% revision surgery was attributed to ARMD. Jennings et al. [3] performed a retrospective study involving 54 MoM-THR surgeries and showed that cup wear triggered by ARMD was the leading cause for revision MoM-THR. Crawford et al. [10] performed a large consecutive series of 188 individuals (203 hips) who were revised for MoM-THR failure and revealed that re-revision which was mostly attributed to aseptic loosening and dislocation was required in 16 hips (7.9%), even though the metal ion levels significantly declined. Moreover, they pointed out that there were noteworthy complications specifically in patients with pseudotumor related to ARMD. Borton et al. [9] reported the outcomes of 180 revisions for failed MoM-THR and showed that cobalt-chromium-containing bearing surfaces are associated with poor functional outcomes.

MoM-THR revised to THR for ARMD had various complications and poor clinical outcomes [23, 24]. However, the timing of MoM-THR revised to THR and the component type of THR have a growing controversy [30]. In the previous reports [31, 32], CTHR is commonly used to revise MoM-THR. Presently, the use of cement-based prosthetics to resist poor bone condition seems to be a trend [32]. Studies [33, 34] assessing early revision of MoM-THR showed superior results before the pseudotumor appeared. Nevertheless, little is known about these results following pseudotumor revision [35].

Several limitations should be acknowledged. First, this is a retrospective analysis with inherent drawbacks. Second, the analysis of variables may be limited by sample size and lack of a control group. Nonetheless, in view of the 10-year follow-up data described following MoM-THR revised to CTHR, we deem that the conclusions drawn from this study are important. Third, our baseline data does not involve the end-organ damage related to ARMD, although there has become an area of growing concern, with the literature [36, 37] describing associated the end-organ damage (i.e., cardiotoxicity), corrosion, and fretting in individuals undergoing CTHR revision. Fourth, we did not measure the blood metal ion levels during patient follow-up, and did not involve the blood metal ion thresholds to predict ARMD, although these thresholds tend to be effective for identifying individuals at low risk of ARMD.

## Conclusions

This study demonstrated CTHR might yield encouraging functional scores and a tolerable rate of key orthopaedic complication. While not presently appreciated in previous reports [8, 17], supplementary evidence will be needed to explicit if ARMD-related wear and loosening are slowed down in the long term with CTHR constructs. In addition, patients with failed MoM-THR revised to CTHR should ponder ARMD-related complications and subsequently the possibility of the need for revision surgery, or balance the potential benefits of improving quality of life against the ARMD-related risks.

## Data Availability

The datasets used and/or analysed during the current study are available from the corresponding author on reasonable request.
